# Health Tract, No. 38

**Published:** 1865

**Authors:** 


### HEALTH TRACT, No. 38.

### HEALTHFUL OBSERVANCES.

1. To eat when you do not feel like it is brutal, nay, this a slander on the lower animals, they do not so debase themselves.

2. Do not enter a sick-chamber on an empty stomach, nor remain as a watcher or nurse until you feel almost exhausted, nor sit between the patient and the fire, nor in the direction of a current of air from the patient toward yourself, nor eat or drink any thing after being in a sick-room until you have rinsed your mouth thoroughly.

3. Do not sleep in any garment worn during the day.

4. Most grown persons are unable to sleep soundly and refreshingly, over seven hours in summer, and eight in winter; the attempt to force more sleep on the system by a nap in the daytime, or a "second nap" in the morning, renders the whole of the sleep disturbed and imperfect.

5. Some of the most painful "stomach aches" are occasioned by indigestion, this generates wind, and hence distension. It is often promptly remedied by kneading the abdomen with the ball of the hand, skin to skin, from one side to another, from the lower edge of the ribs downwards, because the accumulated air is forced on and outwards along the alimentary canal.

6. When you return to your house from a long walk or other exhaustive exercise, go to the fire or warm room, and do not remove a single article of clothing until you have taken a cup or more of some kind of hot drink.

7. In going into a colder atmosphere, keep the mouth closed, and walk with a rapidity sufficient to keep off a feeling of chilliness.

8. Two pair of thin stockings will keep the feet warmer than one pair of a greater thickness than both.

9. The "night sweats" of disease come on towards daylight, their deathly clamminess and coldness is greatly modified by sleeping in a single, loose, long woolen shirt.

10. The man or woman who drinks a cup of strong tea or coffee, or other stimulant, in order to aid in the better performance of any work or duty, public or private, is a fool, because it is to the body and brain an expenditure of what is not yet got; it is using power in advance, and this can never be done, even once, with impunity.

11. The less a man drinks of any thing in hot weather the better, for the more we drink the more we want to drink, until even ice-water palls and becomes of a metallic taste; hence the longer you can put off drinking cold water on the morning of a hot day, the better will you feel at night.

12. Drinking largely at meals, even of cold water or simple teas, is a mere habit and is always hurtful. No one should drink at any one meal more than a quarter of a pint of any liquid, even of cold water, for it always retards, impairs, and interferes with a healthful digestion.

13. If you sleep at all in the daytime, it will interfere with the soundness of your sleep at night much less, if the nap be taken in the forenoon.

14. A short nap in the daytime may be necessary to some. Let it not exceed ten minutes, to this end sleep with the forehead resting on a chair-back or edge of the table.

15. Never swallow an atom of food while in a passion, or if under any great mental excitement, whether of a depressing or elevating character; brutes won't do it

